# Clinical and Magnetic Resonance Imaging (MRI) Features, Tumour Localisation, and Survival of Dogs with Presumptive Brain Gliomas

**DOI:** 10.3390/vetsci9060257

**Published:** 2022-05-27

**Authors:** Marta Pons-Sorolla, Elisabet Dominguez, Michał Czopowicz, Anna Suñol, Christian Maeso Ordás, Carles Morales Moliner, Marc Pérez Soteras, Patrícia Montoliu

**Affiliations:** 1 AniCura Ars Veterinaria Hospital Veterinari, Carrer dels Cavallers 37, 08034 Barcelona, Spain; marta.pons-sorolla@anicura.es (M.P.-S.); e.dominguez@arsveterinaria.es (E.D.); anna.sunol@anicura.es (A.S.); christian.maeso@anicura.es (C.M.O.); carles.morales@anicura.es (C.M.M.); marc.perez@anicura.es (M.P.S.); 2 Division of Veterinary Epidemiology and Economics, Institute of Veterinary Medicine, Warsaw University of Life Sciences—SGGW, Nowoursynowska 159c, 02-776 Warsaw, Poland; mczopowicz@gmail.com

**Keywords:** glioma, epileptic seizures (ES), piriform, dog, Magnetic Resonance Imaging (MRI)

## Abstract

Brain gliomas are common tumours diagnosed in dogs. However, limited information is available on the clinical features and overall survival time (OS) in dogs receiving palliative treatment. The aim of this study was to evaluate possible associations between presenting complaint, tumour localisation, Magnetic Resonance Imaging (MRI) features, survival times, and reason for the death of dogs with suspected intracranial glioma treated palliatively. Sixty dogs from a single institution were retrospectively included (from September 2017 to December 2021). Dogs were included if a presumptive diagnosis of brain glioma was obtained based on an MRI scan and medical history. French Bulldogs were overrepresented (40/60); 46 out of 60 dogs (77%) presented due to epileptic seizures (ES) and in 25/60 dogs (42%), cluster seizures or *status epilepticus* were the first manifestation of the disease. Dogs with suspected gliomas located in the piriform lobe showed a higher probability of presenting due to epilepsy compared to dogs with glioma in other regions, and more frequently died or were euthanised because of increased ES. Magnetic Resonance Imaging (MRI) features differed between localisations. Fronto-olfactory tumours were more frequently, whereas piriform tumours were less frequently, classified as suspected high-grade glioma. The median survival time was 61 days. Dogs with contrast-enhancing suspected gliomas had significantly shorter OS. This study provides additional information on the clinical features and survival of dogs with suspected brain gliomas treated palliatively.

## 1. Introduction

Gliomas are the second most common brain tumour in dogs, after meningiomas [1,2,3]. An overall prevalence rate of 0.9% has been reported, accounting for approximately 30% of primary intracranial neoplasms in dogs [3].

Brachycephalic dogs have shown a predisposition [4,5,6,7,8], and males are reported in many studies to be overrepresented [7,8]. Most brain gliomas are rostrotentorial, and neurological signs are variable, with epileptic seizures (ES) being the most frequently described clinical sign. Histopathologically, gliomas are classified as oligodendrogliomas, astrocytomas, or undefined gliomas, and are further subclassified as low or high grade [8]. Due to financial and safety considerations, tentative antemortem diagnosis of intracranial gliomas is most commonly based on signalment, clinical signs, and compatible magnetic resonance imaging (MRI) findings [9,10].

The reported overall survival times (OS) in canine gliomas are mainly related to the chosen treatment [6,7,11,12]. Described OS for dogs with brain gliomas that receive palliative treatment are short, ranging from 26 to 94 days [7,12,13,14]. However, little is known about possible differences in OS between specific localisations or MRI features in suspected gliomas.

In humans, the predisposition for developing ES depends on the localisation and grade of the tumour [15]. Mesial temporal gliomas are associated with higher epileptogenicity [16]. In addition, low-grade gliomas are more epileptogenic than high-grade gliomas [17,18]. Since low-grade gliomas have an increased affinity for the mesial temporal area in adults [16,19], drug-resistant epilepsy is their most common symptom [17,18]. Investigations of animal models have also indicated that the piriform lobe, located at the edge of the olfactory and mesial temporal cortex, is one of the most epileptogenic regions in the brain [20,21]. Information about the influence of the localisation and nature of the lesion on epileptogenicity of gliomas is very limited in veterinary medicine. Veterinary studies associating localisation of the tumour and MRI features with clinical signs are limited, and most commonly include various types of tumours.

The aim of this study was to evaluate the relationship between tumour localisation and MRI features, as well as presenting clinical signs, and to link these variables with survival times in a population of dogs with suspected intracranial glioma that have undergone a palliative treatment.

We hypothesised that the specific localisation of the suspected glioma in the rostrotentorial region influences presenting clinical signs, MRI features, and survival time. Our main hypothesis was that dogs with suspected gliomas located in the piriform lobe present more frequently due to severe ES compared to other rostrotentorial regions.

## 2. Materials and Methods

### 2.1. Study Design and Medical Records

This retrospective study was carried out including dogs from Anicura Ars Veterinaria veterinary hospital that presented between September 2017 and December 2021. Dogs with a final presumptive diagnosis of brain glioma based on signalment, clinical history, neurological signs, progression, and compatible MRI characteristics were included.

The term “glioma” was searched within the author’s institution’s MRI reports database. All cases with a presumptive diagnosis or main differential diagnosis of glioma in the MRI report conclusions were selected for revision. Complete medical records were reviewed, and the following data were recorded for each case: age, sex, body weight and breed, owner’s main complaint, date of first presentation of clinical signs, physical and neurological examination, type of treatment, overall survival time (time from MRI diagnosis to death, OS), and cause of death or euthanasia when available. When complete information regarding follow up was not included in the medical records, data were obtained by means of a telephone call with the first opinion veterinary or owners.

The findings in the neurological examination were classified by a board-certified neurologist as mild/moderate, severe, or post-ictal following the criteria in Table 1. Post-ictal signs were considered when neurological deficits were symmetrical, resolved in the second neurological examination before additional specific treatment, and the owners reported no behavioural or neurological changes before the presentation.

For dogs with ES, information about the type of ES (focal or generalised) and severity (isolated ES or cluster seizures/*status epilepticus* (CS/SE)) was recorded. Clustered ES or *status epilepticus* were included in the same group due to the lack of specific information in some clinical history records to differentiate between them. It was also recorded whether CS/SE was the first manifestation of neurological disease.

*Status epilepticus* and CS were defined following the International Veterinary Epilepsy Task Force Consensus proposal. *Status epilepticus* was defined clinically as (a) an ES lasting more than 5 min or (b) two or more discrete ES between which there is incomplete recovery of consciousness. Cluster seizures were defined as two or more seizures within a 24-h period [22].

When available, additional diagnostic tests including cerebrospinal fluid (CSF) analysis, thoracic radiographs, abdominal ultrasound, or computed tomography (CT) were reviewed for the presence of signs of neoplasia or severe systemic disease.

Dogs were excluded if they showed other neoplasia or severe systemic disease that could influence neurological signs or OS. Dogs that received chemotherapy or radiotherapy, dogs that died or were euthanised <24 h after MRI without any intent of treatment, and dogs with a caudotentorial localisation were included in the demographic study but excluded from the survival analysis. Dogs that were alive at the end of the study, died of conditions unrelated to suspected glioma, and dogs whose date of death was unknown were censored.

### 2.2. MRI and Tumour Localisation

All MRI scans were performed under general anaesthesia using a high-field 1.5T MRI scanner (Vantage Elan, Canon Medical Systems Corporation, Tochigi, Japan). The most common MRI protocol used included T2-weighted (T2W) sagittal and dorsal planes, transverse planes of T2W, T1-weighted (T1W), T2 fluid-attenuated inversion recovery (FLAIR), and dorsal and transverse planes of T1W post-gadolinium sequences (Table S1). Some patients had additional sequences including transverse diffusion-weighted echo-planar sequences, T2-weighted gradient echo (T2*), or susceptibility-weighted images. For post-contrast images, gadoteridol (Clariscan^®^-GE Healthcare AS, Oslo, Norway- injection 0.5 mmol/mL) was administered at a dose of 0.2 mL/kg intravenously (IV). The terms presumptive and suspected are used in the study since histopathological confirmation of diagnosis was not available.

All MRI images were reviewed for the purpose of the study by one board-certified radiologist (E.D.) and one board-certified neurologist (P.M.) who were blinded to the initial imaging report and patient’s clinical signs. 

MRI features were considered consistent with glial tumours when intra-axial single lesions, often heterogeneous, ovoid to amorphous, well-defined to infiltrative, T1 isointense to hypointense, and T2 isointense to hyperintense mass lesions with variable contrast enhancement (CE) were observed. Other considered findings were cyst-like lesions, intralesional haemorrhage, vasogenic oedema, and mass effect [23].

Additional criteria including clinical history, neurological signs, case follow-up, CSF, and follow-up MRI, if available, were reviewed at a later stage in the case of diagnostic discordance between the imaging report and the review performed by the board-certified radiologist and neurologist. Cases initially diagnosed as most consistent with glioma were excluded if no consensus was reached after reviewing the images. Patients with severe concomitant brain lesions or other lesions that could cause ES or affect the survival time were excluded from the study.

For each case included, the following MRI features were recorded: localisation, lateralisation, margins, cavitation, presumed invasiveness, and invaded areas, presumed invasion or oedema of the hippocampus, predominant MRI signal intensities in T1W, T2W, and FLAIR sequences, presence of signal void on T2* sequences, presence of abnormalities in the bones adjacent to the lesion, presence of suspected perilesional and/or vasogenic oedema, CE, presence of suspected post-ictal changes, and MRI features evaluating mass effect (subarachnoid CSF effacement, midline shift, ventricular compression/displacement, and brain herniation). Additionally, a board-certified radiologist (E.D.), who was blinded for any clinical information, was asked for a subjective evaluation of tumour grade given the overall MRI features with three options: low-grade, high-grade, or equivocal. High-grade gliomas were suspected when the lesions had ill-defined margins, demonstrated the heterogeneous signal intensity and CE, and showed signs of internal haemorrhage, cavitation, and/or ventricular/intraventricular extension. Low-grade gliomas were suspected when the lesions had better-defined margins, an overall more uniform signal intensity, and an absence of CE. Low-grade and equivocal groups were later merged into “non-high-grade” for meaningful statistical analysis. To ensure comparable subjective grading, images were reviewed consecutively for three days.

Suspected gliomas were divided into groups based on the localisation of the majority of the volume of the lesion as follows: fronto-olfactory, piriform, temporal, parietal, occipital, intraventricular, diencephalic, and caudotentorial (brainstem or cerebellum).

### 2.3. Statistical Analysis

Numerical variables were presented as a median, interquartile range (IQR), and range, and compared between two groups using the Mann–Whitney U test and between >2 groups with the Kruskal–Wallis H test, followed by Dunn’s post-hoc test. Categorical variables were presented as counts and percentages and compared between groups using the maximum likelihood G test or Fisher exact test if a count in any cell of the contingency table was <5. The 95% confidence intervals (CI 95%) for proportions were calculated using the Wilson score method. The relationship of demographic and clinical characteristics with the localisation in the central nervous system was investigated first in the univariable analysis and variables with *p* < 0.1 were entered into the multivariable multinomial logistic regression (Table S7). For the multivariable analysis, tumour localisation was merged into three categories: fronto-olfactory, piriform, and other rostrotentorial localisations. The relationship of demographic and clinical characteristics with the high-grade was investigated first in the univariable analysis, and variables with *p* < 0.1 were entered into the multivariable binary logistic regression. The strength and direction of the relationship were expressed using the odds ratio (OR). The univariable and multivariable Cox proportional hazard models were used to investigate the relationship of demographic and clinical characteristics with the overall survival time (OS) and to estimate the hazard ratio (HR). Survival curves were presented for significant variables using the Kaplan–Meier product-limit estimator. Survival analysis included only dogs with rostrotentorial suspected gliomas treated palliatively and only glioma-related deaths were considered complete records. Dogs were censored if: (1) they were still alive when the final database was built, (2) they died of conditions unrelated to suspected glioma, or (3) the date of death was unknown (in this case, the date of the last follow-up was used as the date of censoring). All multivariable analyses were carried out according to the backward stepwise procedure. A significance level (α) of 0.05 was assumed in all statistical tests except for the univariable analyses in which α was 0.1. The statistical analysis was performed in TIBCO Statistica 13.3 (TIBCO Software Inc., Palo Alto, CA, USA) and IBM SPSS Statistics 26 (IBM Corporation, Armonk, NY, USA).

## 3. Results

### 3.1. Demographics

In the study period, 85 dogs with MRI consistent with suspected glioma were eligible for inclusion in the study. In total, 25 dogs were excluded due to any of the following reasons: equivocal MRI diagnosis with other suspected brain tumours, vascular accidents, inflammatory diseases, or post-ictal changes (18 dogs); clinical progression not consistent with brain neoplasia (2 dogs); concomitant congenital hydrocephalus that may have affected OS and clinical signs (2 dogs); lack of clinical information (2 dogs); and abdominal effusion and suspected lymphoma (1 dog).

Sixty dogs met the inclusion criteria. There were 32 males (19/32 neutered) and 28 females (23/28 spayed). The male-to-female ratio was 1.14 (CI 95%: 0.69–1.89). Age at presentation ranged from 4 to 15 years with the median (IQR) of 9 (7–10) years. Body weight ranged from 4 to 55 kg with the median (IQR) of 14 (12–18) kg. Breeds included French Bulldogs (40 dogs), Boxers (6 dogs), mixed breed dogs (5 dogs), Yorkshire Terrier (2 dogs), and one each of the following breeds: English Bulldog, Boston Terrier, Cavalier King Charles Spaniel, West Highland White Terrier, Staffordshire Bull Terrier, Cane Corso, and Dogue de Bordeaux. Detailed demographic characteristics of the study population are presented in Table S2.

### 3.2. Clinical Presentation and Clinicopathologic Findings

The time from the first signs to diagnosis ranged from 1 to 730 days with the median (IQR) of 13 (3–35) days. The diagnosis was made within 2 weeks of presentation of clinical signs in 50% of dogs, and within 5 weeks in 75% of dogs.

Epileptic seizures were the most common reason for presentation, recorded for 46/60 dogs (77%; CI 95%: 65–86%). Thirty-four of sixty dogs (57%; CI 95%: 44–68%) had CS/SE. In 25/60 dogs (42%; CI 95%: 30–54%), CS/SE was also the first manifestation of the disease. Epileptic seizures were described by the owner as being generalised tonic-clonic in 36/46 dogs (78%), 8/46 dogs (17%) had suffered both from focal and generalised tonic-clonic ES, and 2/46 dogs had shown only focal ES. In only 8 of 46 dogs (17%) that presented due to ES, other neurological signs (obtundation, circling, weakness, or altered behaviour) were also described by the owner.

A total of 13 of 60 dogs (22%; CI 95%: 13–34%) presented due to neurological signs without a history of ES. In one dog, suspected glioma was considered an incidental finding, and MRI was performed due to an acute onset of facial paralysis, which was considered idiopathic.

On neurological examination, 31/60 dogs (52%; CI 95%: 39–64%) had neurological deficits and in only 1 dog they were classified as severe. Neurological examination was normal at the time of presentation or deficits were considered post-ictal in 29/60 (48%) dogs. 

Results of CSF analysis were available for review in only 6 dogs (10%). Mild pleocytosis was observed in 2 dogs and albumin-cytological dissociation in the other 2 dogs.

### 3.3. MRI Features and Localisation of Suspected Glioma

**Figure 1:**
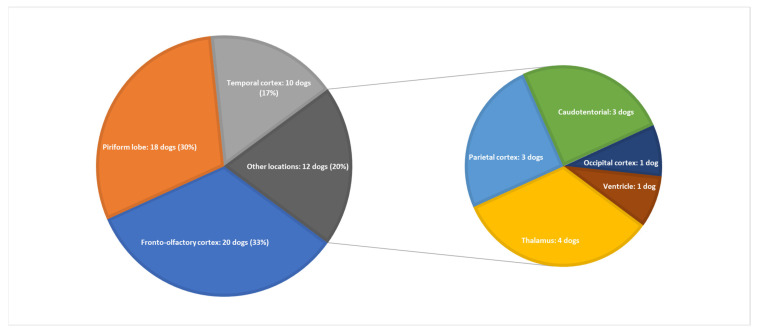
Suspected glioma location in the 60 dogs studied.

Most suspected gliomas (48/60 dogs; 80%) were located in the fronto-olfactory cortex (20/60 dogs; 33%), piriform lobe (18/60 dogs; 30%), and temporal cortex (10/60 dogs; 17%). The remaining 12 presumptive gliomas (20%) were located in the thalamus (4 dogs), parietal cortex (3 dogs), occipital cortex (1 dog), and lateral ventricle (1 dog), or were caudotentorial (3 dogs). (Figure 1). Detailed clinical characteristics of the study population are presented in Table S2.

Tumour invasion or oedema of the hippocampus was observed in 15/18 dogs with piriform lobe suspected gliomas, 1 dog with the tumour in the temporal cortex, and 1 dog with an intraventricular presumptive glioma. Detailed MRI features of the study population are presented in Table S3.

Additionally, 9 dogs underwent a second MRI scan, reaffirming the suspected diagnosis of brain tumour consistent with glioma in 8 of them. One dog had a second MRI performed after radiotherapy treatment.

### 3.4. Association among Suspected Glioma Localisation, Demographic Data, Clinical Features, and MRI Features

**Figure 2:**
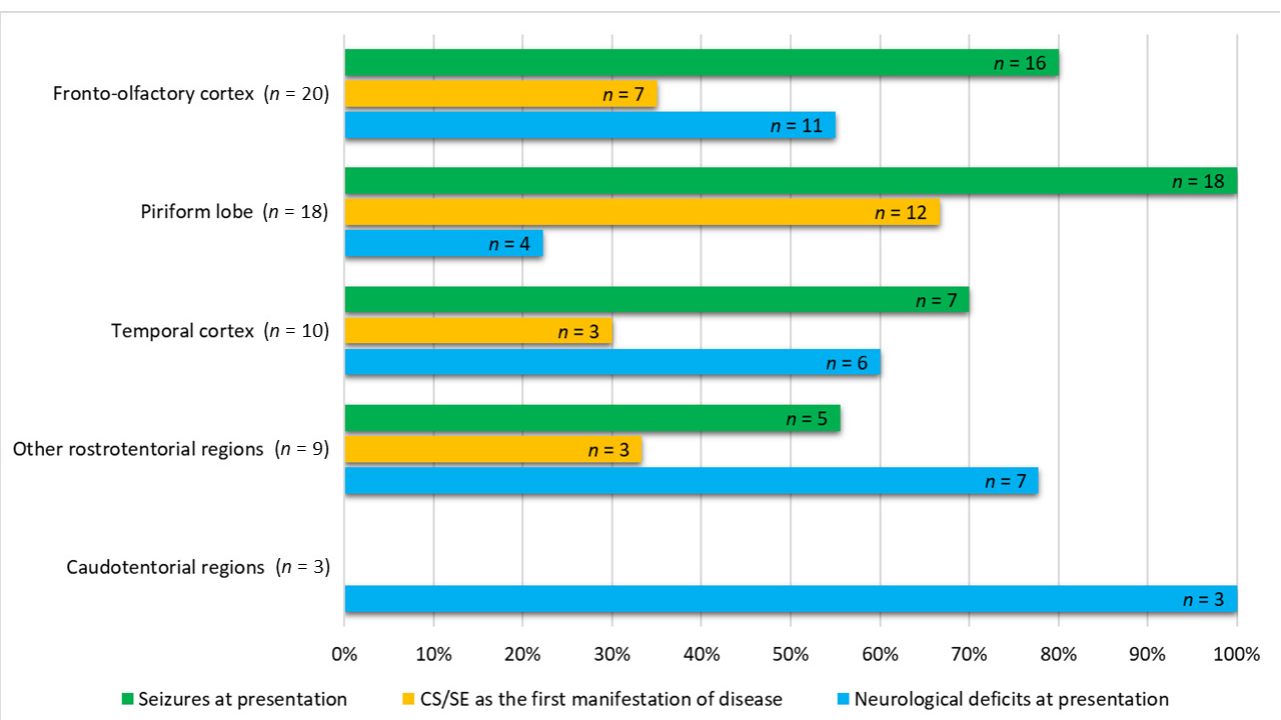
Comparative numbers of cases out of 60 dogs studied having seizures, cluster seizures/*status epilepticus* (CS/SE), as the first manifestation of disease, and presence of neurological deficits not considered post-ictal at admission.

In the univariable analysis of variables associated with glioma location, suspected gliomas in the fronto-olfactory localisation were significantly more often diagnosed in females (*p* = 0.003), and dogs with presumptive gliomas in this localisation were significantly lighter than dogs with gliomas in other localisations (*p* = 0.029). In dogs with suspected gliomas in the piriform localisation, ES were observed significantly more often (*p* = 0.010), and neurological deficits were observed significantly less often (*p* = 0.024) compared to dogs with suspected gliomas in other localisations (Table S4; Figure 2). In the multivariable analysis, two variables remained significantly and independently related to the tumour localisation: fronto-olfactory localisation was more common in females (*p* = 0.011), and ES were more common in tumours located in the piriform lobe (*p* = 0.006).

**Figure 3:**
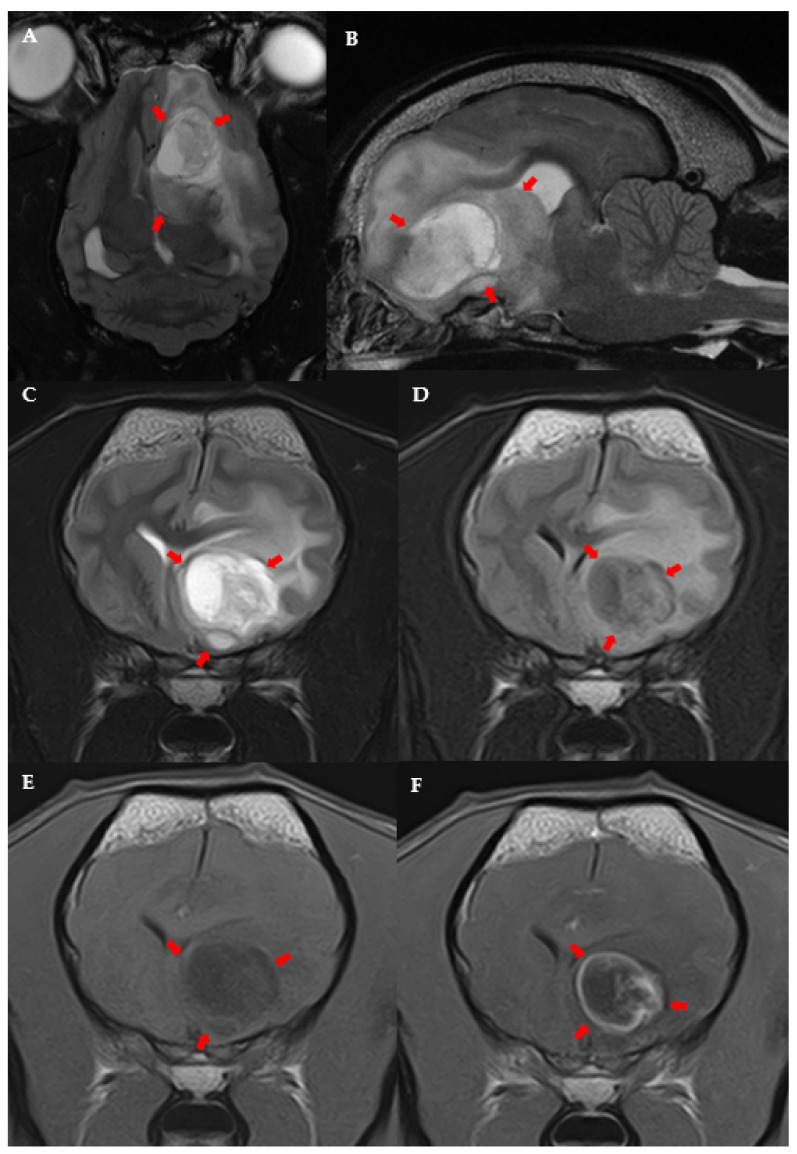
MRI images of suspected high-grade glioma in the left fronto-olfactory cortex in an 8-year-old female French Bulldog: Dorsal (**A**) and left parasagittal (**B**), T2-w. Transverse T2-w (**C**), T2-FLAIR (**D**), T1-w (**E**), and T1 post-contrast (**F**). The left side of the patient is on the right side of the image. Arrows point to the lesion.

**Figure 4:**
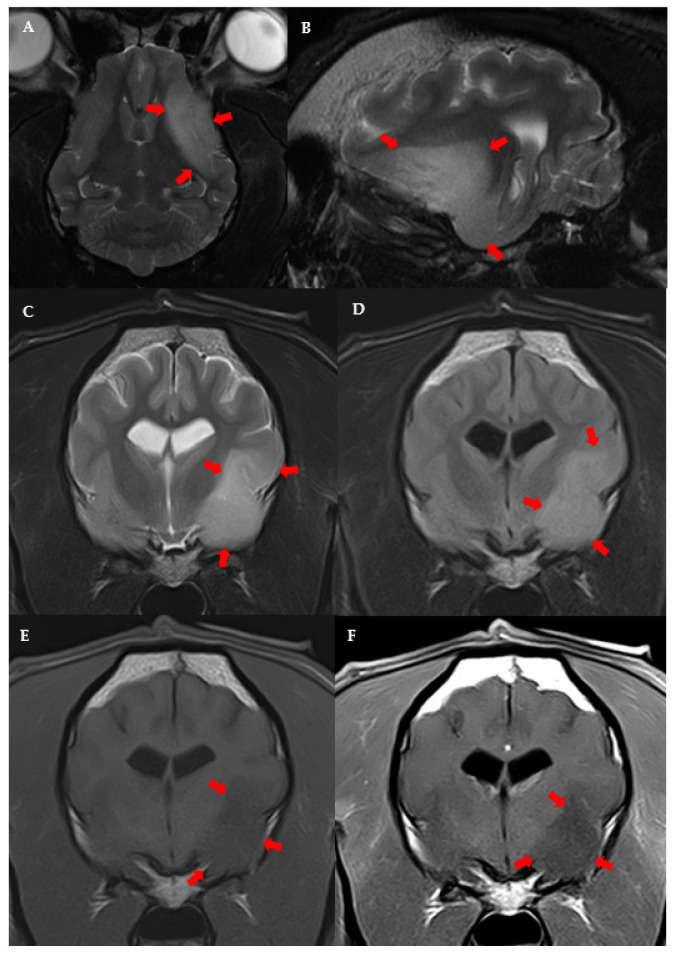
MRI images of suspected low-grade glioma in the left piriform lobe of a 9 year-old male French Bulldog. Dorsal (**A**) and left parasagittal (**B**), T2-w. Transverse T2-w (**C**), T2-FLAIR (**D**), T1-w (**E**), and T1 post-contrast (**F**). The left side of the patient is on the right side of the image.

The following variables were significantly linked to the MRI diagnosis of high-grade presumptive glioma in the univariable analysis (Table S5): high-grade suspected gliomas were less often observed in large breed dogs (*p* = 0.036); neurological deficits at presentation were more frequent in dogs with suspected high-grade glioma (*p* = 0.038); and suspected high-grade gliomas were more often located in the fronto-olfactory cortex (*p* < 0.001) and less often located in the piriform lobe (*p* < 0.001). In the multivariable analysis, only localisation of the suspected tumour remained significantly associated with presumed glioma grade: tumours in the fronto-olfactory cortex were significantly more often (OR = 9.00; CI 95%: 1.67–48.4; *p* = 0.011) classified as suspected high-grade gliomas and tumours in the piriform lobe were significantly less likely (OR = 0.13; CI 95%: 0.02–0.68; *p* = 0.016) to be considered high-grade, compared to tumours located in other regions (Figure 3 and Figure 4). The multivariable model fit the data well (Hosmer-Lemeshowchi-squared H&L χ^2^ = 0.00, *p* = 0.999; Nagelkerke’s pseudo-R^2^ coefficient = 0.48).

### 3.5. Treatment

All dogs received prednisolone (Prednicortone^®^, AE Bladel, Netherlands) at an initial dose ranging from 0.5–1 mg/kg/24 h, gradually tapered based on neurological signs and/or undesirable side effects. All patients presenting ES received anticonvulsant treatment, either as monotherapy or as a combination therapy with one or more of the following medications: phenobarbital (Soliphen^®^, Pont du Chateau, France), levetiracetam (Keppra^®^, Brussels, Belgium), and potassium bromide (Libromide^®^, Bladel, The Netherlands). Anticonvulsant drugs were maintained long-term until death or euthanasia and drugs and doses were adapted to ES frequency and severity. When increased intracranial pressure was suspected based on neurologic examination or MRI findings, one or more mannitol (Manitol 20% Braun^®^, Barcelona, Spain) intravenous bolus of 0.3–0.5 g/kg were administered. Four animals received a definitive treatment—chemotherapy (one dog receiving temozolamide (Temodal^®^, Harlem, The Netherlands) and one lomustine (CeeNU^®^, Oslo, Norwey) or radiotherapy (two dogs).

### 3.6. Survival Time and Cause of Death

A total of 48 of the 60 dogs (80%) were included in the survival analysis. The remaining 12 dogs were excluded because of one of the following reasons: euthanasia immediately after diagnosis without any attempt for treatment (4 dogs), application of a definitive treatment (4 dogs), caudotentorial localisation of suspected glioma (3 dogs), or lack of follow-up after 12 h from diagnosis (1 dog). Complete information on death related to suspected glioma was available for 37 of 48 dogs (77%). The remaining 11 dogs were censored because they were still alive at the end of the study period (6 dogs), the exact date of their death was unknown (4 dogs), or the cause of death was unrelated to glioma (1 dog).

**Figure 5:**
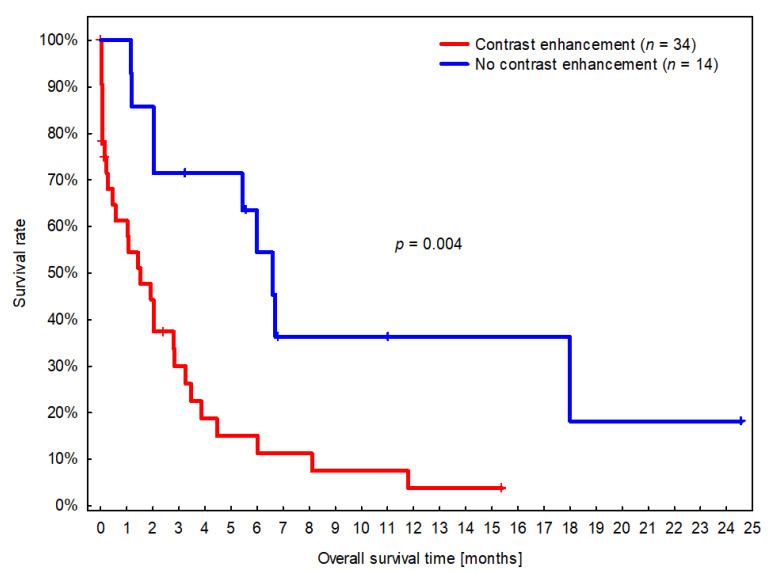
Kaplan–Meier survival curves for dogs with presumed glioma with and without contrast enhancement (CE). Short vertical lines signify censored dogs.

The median overall survival time (OS) was 61 days (CI 95%: 32–89 days; IQR: 15–181 days). In the univariable analysis, four variables were significantly associated with OS: Large dogs survived longer (HR = 0.3; CI 95%: 0.1–0.9; *p* = 0.031), whereas OS was shorter for dogs with presumptive gliomas in the fronto-olfactory region (HR = 3.0; CI 95%: 1.5–5.9; *p* = 0.002), for dogs with contrast-enhancing presumptive glioma (HR = 3.2; CI 95: 1.5–7.2; *p* = 0.004), and dogs with MRI-based high-grade glioma (HR = 2.1; CI 95: 1.1–4.2; *p* = 0.032). The only variable that remained significant in the multivariable analysis was CE. Dogs with suspected gliomas with CE had significantly shorter MST (46 days, CI 95%: 10–81 days) compared to dogs with non-CE gliomas (198 days, CI 95%: 159–237 days) (Figure 5).

**Figure 6:**
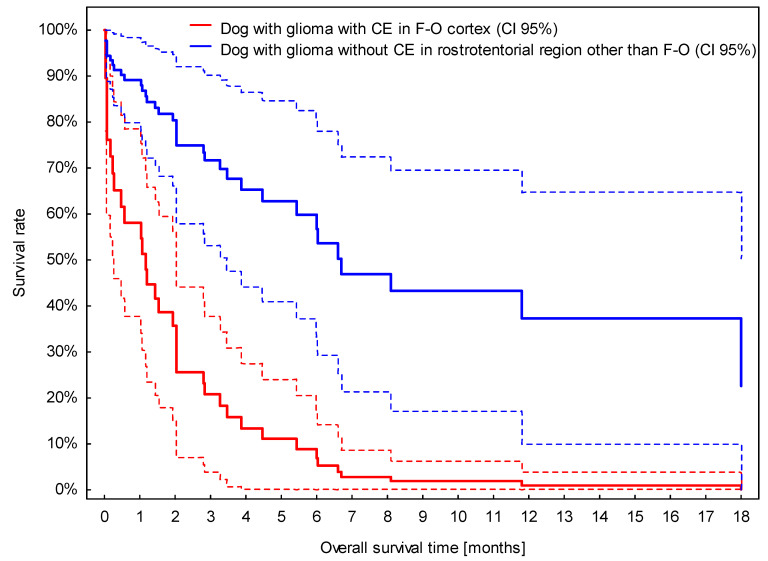
Survival curves for dogs with presumed glioma located in the fronto-olfactory cortex (F-O) with contrast enhancement (CE) and located in other rostrotentorial regions without CE. Broken lines stand for 95% confidence intervals (CI 95%) for estimated survival curves.

The presence of CE was associated with a roughly three-times higher risk of death (Table S6). Although the location of suspected glioma in the fronto-olfactory cortex was removed from the multivariable analysis due to a *p*-value slightly above 0.05 (*p* = 0.071), the model based on CE and location in the fronto-olfactory cortex may play a prognostic role, as dogs with presumptive glioma located in the fronto-olfactory cortex and with CE survived significantly shorter than dogs with suspected glioma in different locations and without CE (Figure 6).

The cause of glioma-related death was known for 32/48 dogs with suspected glioma in the rostrotentorial localisation, including 30 dogs with palliative treatment, 1 dog receiving temozolamide, and 1 dog that underwent radiotherapy. In total, 3 of 32 dogs (9%) were euthanised due to a lack of improvement before hospital discharge during the first 48 h. Thirteen dogs (41%) died or were euthanised mainly due to the progression of neurological signs. In 16 dogs (50%), ES were the main reason for death or euthanasia. When evaluating the association with tumour localisation (Table 2), euthanasia related to ES was significantly more often observed in dogs with suspected glioma in the piriform lobe (*p* = 0.013).

## 4. Discussion

In the present study, we provide additional and extended information about the clinical features of presumed brain gliomas in dogs, describing differences in the clinical picture depending on their localisation in the rostrotentorial region. We also described differences in OS depending on MRI features, and the cause of death or euthanasia is evaluated depending on tumour localisation.

### 4.1. Clinical and MRI Features and Tumour Location

Previous studies have found a genetic predisposition to develop brain gliomas in Bulldogs, Boxers, and Boston Terriers, with percentages ranging from 48% to 78% [4,5,6,7,8,10,12,24]. In our study, 80% of dogs belonged to one of these breeds. Our population differed from others in that two-thirds of the dogs were French Bulldogs, while in other reports these percentages ranged between 7% and 52% [5,6,7,24]. This probably not only reflects the popularity of this breed in our geographical area but also highlights a higher predisposition. The median body weight in our study was 14 kg, although larger dogs have been shown to suffer from brain tumours more often in previous reports [2,6,25,26]. This is most likely due to the overrepresentation of French bulldogs in our study. However, we found differences in OS between dog sizes, with a longer OS for heavier dogs. Although this may be influenced by the low number of large dogs included in our study, and it did not hold in the multivariable analysis, further investigation is needed to determine the role of breed or body size in glioma location and prognosis.

The median age for all dogs at the time of diagnosis was 9 years of age, which is consistent with previous studies [5,7,11,12,13,25]. Other studies report a male predisposition for brain gliomas in dogs [7,8,11], similar to that described in humans [18,27]. In our study, the overall male/female ratio was 1.14. Surprisingly, gliomas in the fronto-olfactory localisation were significantly more often diagnosed in females in our study.

The most common clinical sign associated with brain tumours in dogs are ES [3,28,29,30]. Reported structural epilepsy ranges from 50% to 75% in studies where all types of brain tumours are included [3,14,29,30]. References including only suspected or confirmed brain gliomas report the occurrence of ES in 62% to 92% of dogs [5,7,12,13]. This is in accordance with our study, where 77% of patients presented due to ES.

Little information is available in the veterinary bibliography regarding the type of ES (focal, generalised, or focal with secondary generalisation) in dogs with brain tumours. Our results are consistent with most previous studies which describe generalised ES as being more common than focal [7,29,30]. However, the retrospective nature of studies may underestimate focal seizure onset, as focal origin may often be clinically inapparent, and prospective studies including more detailed anamnesis are lacking.

The reported prevalence of CS/SE in brain tumours is variable. In one study, 43% of dogs with brain gliomas had CS [7]. However, in a recent study including different types of rostrotentorial brain tumours, only 9% of dogs developed CS and 8% SE [30]. In our study, 34 dogs (74% of dogs with ES) suffered from CS/SE. Interestingly, for 25 of those cases, CS/SE was the first manifestation of neurological disease. This data deserves, in our opinion, further investigation to evaluate if different tumour types differ in frequency of CS/SE as the first manifestation of disease.

Seizure development secondary to brain tumours has a complex mechanism which is not completely understood [31]. Several explanations have been suggested, including physical distortion of the brain parenchyma, neurotransmitter imbalance (decrease in inhibitory and increase in excitatory neurotransmission), vascular compromise (ischaemia), denervation in cortical areas, structural reorganisation, functional differentiation with the peritumoral neocortex (synaptic alterations, neuronal and glial loss, or reactive astrogliosis) and immunological alterations [18,31,32,33]. Moreover, molecular factors have been associated with epileptogenesis in brain tumours in humans. Low-grade gliomas have a mutation in two codons of isocitrate dehydrogenase (IDH). The IDH1 mutation is more prevalent in low-grade gliomas and causes the formation of 2-hydroxyglutarate instead of alfa-ketoglutarate. As 2-hydroxyglutarate has a structural similarity with glutamate, activation of N-metil-D-aspartate receptors (NMDA) and increased neuroexcitatory activity occurs [17,34,35].

Seizure activity is known to originate mainly from cerebral hemispheres [36]. However, information is lacking in veterinary medicine regarding specific rostrotentorial areas associated with higher epileptogenicity. One study suggested that a higher incidence of ES was found secondary to tumours in the temporal, frontal, parietal, or olfactory cortex [37]. Another study concluded that dogs with neoplastic tissue in the frontal lobe were at a higher risk of developing ES than in other localisations [29]. In our study, localisation in the piriform lobe was independently associated with a higher probability of ES. All dogs with lesions located in the piriform lobe suffered from ES, compared to 80% of dogs in the fronto-olfactory cortex, 70% of dogs with lesions in other areas of the temporal cortex, and 56% of dogs with other rostrotentorial lesions (Figure 2).

There are two main anatomically related cortical areas that have been associated with more frequent and severe ES in human medicine—the piriform lobe and the mesial area at the temporal cortex [38,39,40]. The piriform cortex has been described as a seizure activity generator and focal seizure amplificatory [41,42]. Although the exact mechanism is not completely understood, two specific deep piriform areas (the piriform cortex and area tempestas) have been defined as kindling seizure areas [38]. Moreover, the piriform cortex has broad connections with other brain regions [43] and has been suggested to play a critical role in seizure propagation [20]. Gliomas located in the mesial temporal area are associated with a higher incidence of ES compared to frontal or parietal localisations in humans [41,44]. The mesial temporal area includes the temporal amygdala, entorhinal area, allocortex, mesocortex, and hippocampus [45]. The hippocampus is considered a highly epileptogenic area in humans [21,42,46,47], and structural alterations at the hippocampus have been linked to idiopathic epilepsy and temporal lobe epilepsy in veterinary medicine as well [21]. The hippocampus and piriform lobe share anatomical characteristics, including the presence of three excitable neuronal layers, and similar structural changes are observed in both localisations due to sustained neuronal injury in humans with ES [39,42] suggesting common epileptogenic mechanisms for both areas. In this study, hippocampal affection by the tumour was almost exclusively observed in presumed gliomas affecting the piriform lobe, which may have also played a role in seizure generation. Due to this fact, it was also impossible to discriminate between the role of piriform location and hippocampus involvement in epileptogenesis.

In previous studies [3,7,9,29], different criteria were used to classify the localisation of tumours. In this study, we initially grouped suspected gliomas considering major brain and cortical divisions. Areas included were the cerebral cortex (fronto-olfactory, parietal, temporal, piriform, and occipital), diencephalon, intraventricular, and caudotentorial, similar to another recent study [7]. However, the piriform lobe is considered part of the temporal cortex in this study [7] and other previous reports, which may explain discrepancies with our results. We decided to separate lesions located in the piriform lobe, as we hypothesised, based on our clinical experience and human bibliography [20,38,40,41,42], that piriform lobe localisation could be related to the higher frequency of structural epilepsy and lower frequency of neurological deficits [38,40,41,42,48]. In the human bibliography, the piriform cortex is occasionally considered a region of the mesial temporal cortex [41], however, most authors include the piriform lobe as part of the olfactory cortex [20,49].

Thirty-one dogs (52%) showed neurological signs at presentation that were interpreted to be a consequence of a structural brain lesion. In the other 48% of cases, the neurological exam was normal, or deficits were considered post-ictal. Other studies reported a variable absence of neurological deficits between 5% and 60% [5,6,30]. Interestingly, a considerable proportion of dogs had deficits on neurological examination while no other neurological signs had been observed by owners. This emphasises the importance of a complete neurological examination in dogs with ES. Neurological signs in our study were mostly considered mild/moderate. Rostrotentorial localisations that were less commonly associated with neurological deficits were in the piriform lobe (only 22% of dogs showed neurological deficits) and fronto-olfactory cortex (55% of dogs showed deficits). The fronto-olfactory cortex and piriform lobe have been previously described as “clinically silent regions” [40]. However, to the best of the author’s knowledge, no studies have investigated in detail the prevalence of neurological deficits depending on the specific brain area affected. A possible explanation is that dogs with piriform lobe tumours may be diagnosed before due to ES occurrence early in the course of the disease. Low-grade slow-growing gliomas have been described in humans to most frequently produce ES without additional neurological deficits, in contrast to higher-grade faster-growing tumours [7,33].

Gliomas are histopathologically classified as oligodendrogliomas, astrocytomas, or undefined gliomas, and further subclassified as low- or high-grade [8]. In the present study, we did not attempt to classify tumour subtypes based on MRI features. A low correlation with histopathological diagnosis has been described, and glioma subtype determination has a low clinical relevance regarding treatment and prognosis. The accuracy of tumour grading in cases of suspected glioma based on MRI features is also only moderate. An accuracy of 53% for predicting tumour grade was observed in one study [24], and another study reported a sensitivity of 67% and specificity of 57% for the diagnosis of a high-grade tumour [7]. Described MRI features associated with lower-grade tumours include mild to no CE, absence of cystic structures, and tumour localisation other than the thalamo-capsular region. Features associated with high-grade gliomas include spread to neighbouring brain structures and presence of CE [7,9,10]. We decided to classify a suspected high-grade group based on MRI features, as this is the most common scenario in clinical practice.

In this study, the suspected grade of glioma was significantly related to the localisation. Lesions in the fronto-olfactory cortex were more often classified as suspected high-grade gliomas, while lesions in the piriform lobe were less frequently considered high-grade. It is likely that, similar to humans, low-grade gliomas in dogs may locate preferentially in the piriform lobe and invade mesial temporal areas [16,19,20]. However, this is not supported by previous studies in dogs based on a histopathological diagnosis of tumours, although in these previous studies, the piriform lobe was not considered separately [5,12,29]. Further studies evaluating glioma grade with histopathological confirmation are needed to evaluate if low-grade gliomas are located preferentially in specific brain areas, as is suggested in our study, and occur in humans [16,19].

### 4.2. Survival Time

The median survival time from diagnosis in our study was 2 months with a precision of ±1 month (CI 95% from 32 to 89 days). Few studies have evaluated the survival of dogs with brain gliomas treated palliatively, and variable survival times between one and three months have also been described [7,12].

The median time from first clinical signs to diagnosis was 13 days, and in 75% of dogs, the diagnosis was made within 5 weeks of the first manifestation of the disease. However, the time to diagnosis ranged up to 730 days. This must be interpreted with caution. It is likely that the initial clinical signs in some of these dogs were not related to suspected glioma. For example, a dog with idiopathic epilepsy may have been referred due to increasing seizure frequency or development of new neurological signs, but the time of onset of the first seizure was recorded as the first clinical sign. On the other hand, it is possible that some of these presumptive gliomas were very slow-growing, and the period of time between the first manifestation of the disease, and referral for diagnosis, might have been longer. Unfortunately, due to the retrospective nature of the study, it is not possible to discern between these hypotheses for each case.

In this study, dogs with suspected gliomas with contrast enhancement had significantly shorter MST (less than 3 months), compared to dogs with non-contrast enhancing tumours (5 to 8 months) (Figure 5). Surprisingly, the association with OS appeared stronger for CE than for MRI-based high-grade categorisation. Contrast enhancement was one of the criteria used to classify tumours as high-grade in this study, as has been reported before [9,10,24]. Nevertheless, this is a remarkable conclusion, because as mentioned above, sensitivity and specificity for MRI-based grading have been shown to be low to moderate and therefore less useful as a prognostic indicator in the clinical scenario where biopsies are rarely available [7,25].

In humans, high-grade gliomas are associated with shorter OS than low-grade gliomas [34]. However, in dogs, it is likely that features other than tumour grade also influences survival time. Refractory structural epilepsy is a frequent cause of euthanasia in dogs with brain tumours, and low-grade gliomas located at epileptogenic areas may therefore be associated with shorter OS.

The fronto-olfactory cortex was related to shorter OS in the univariable analysis. Although removed from the multivariable analysis due to a *p*-value slightly above 0.05 (*p* = 0.071), the combination of CE and fronto-olfactory cortex may be somehow useful for prognosis. This is probably related to the fact that lesions in fronto-olfactory localisation were more often classified as suspected high-grade gliomas. Caudotentorial tumour localisation has been previously shown to be associated with a worse prognosis in all types of brain neoplasia [14]. Few studies have investigated differences in survival time between rostrotentorial and caudotentorial localisation specifically for gliomas, and no significant differences were found [7,12]. However, to the best of the author’s knowledge, no previous studies are available evaluating the possible differences in survival time depending on the specific rostrotentorial region involved.

Overall survival time was not associated with the occurrence of ES at presentation in this study. This contrasts with another study, where dogs presented with ES showed longer OS [7]. However, in our study, structural epilepsy was the main tumour-related clinical factor contributing to euthanasia. Epileptic seizures were the main reason for death or euthanasia in half of the dogs for which the information was available. This is consistent with previous studies including all types of tumours, where between 33% and 71% of dogs died or were euthanised because of seizure activity [14,50]. However, this has not been investigated before for a specific tumour type. Furthermore, this association was especially observed for suspected gliomas in the piriform lobe. Of the 11 dogs with suspected piriform lobe glioma whose cause of death was known, 10 were euthanised due to seizure activity (91%). This result suggests that dogs with suspected gliomas in the piriform lobe may require more aggressive antiepileptic treatment, whereas in other localisations, more conservative strategies may be adopted to minimise sedation as an adverse effect. In our opinion, this warrants further investigation, and prospective studies evaluating the effect of individualised antiepileptic treatment strategies based on tumour location would provide useful clinical information on the palliative management of suspected brain gliomas.

### 4.3. Limitations

The main limitations of this study include its retrospective nature and the lack of histopathological confirmation of the clinical diagnosis of glioma. We cannot, therefore, completely rule out that some of the lesions categorised as gliomas were in fact other tumours or even of non-neoplastic nature. The grade of tumour was established by a board-certified imaging specialist, based on previously reported MRI features of high-grade glioma, although only moderate accuracy for grading has been described [8,10]. However, we believe this reflects the clinical challenge of everyday practice. Because of the high cost and risks, brain biopsies and follow-up MRIs are rarely performed in dogs with brain lesions, and clinical decisions are mostly based on imaging findings and the dog’s clinical condition. Moreover, initial tumour grading into suspected high-grade, low-grade, and equivocal had to be re-classified into suspected high-grade and non-high grade for meaningful statistical analysis due to the low number of cases.

Another limitation is the lack of consideration of some suspected glioma MRI descriptive criteria in the data analysis. We did not investigate if tumour/brain volume ratio or other MRI features such as perilesional oedema or intralesional haemorrhage were associated with neurological signs or survival. Future studies including an increased number of cases and all these parameters would be interesting to further characterise the relationship between MRI features and localisation with neurological progression and survival times in dogs with suspected brain glioma.

Due to the overrepresentation of French Bulldogs, our results may not be fully representative of the general canine population. Some of the patients were lost to follow-up and some were still alive at the end of the study, which may have also influenced survival times. A decision was made to merge clusters of seizures and *status epilepticus* in one class (CS/SE). Cluster seizures are defined as two or more seizures within a 24-h period, while *status epilepticus* is defined as an ES lasting more than 5 min, or two or more discrete ES between which there is incomplete recovery of consciousness [36]. However, due to the retrospective nature of the study, information about the complete recovery between ES was not accurate enough in some cases to reliably differentiate between them. Moreover, it is possible that more dogs developed CS/SE during the course of the disease, which was not recorded due to the retrospective nature of the study. Furthermore, palliative treatment was not standardised. Dogs included in this study received different anti-epileptic drugs and at different doses based on clinical decisions. This may have influenced survival times and cause of death.

## 5. Conclusions

Cluster seizures or *status epilepticus* were a frequent first manifestation of disease in dogs with suspected brain glioma. Localisation of the tumour influenced presenting clinical signs and cause of death: dogs with suspected gliomas in the piriform lobe presented more frequently due to ES and were more likely euthanised due to poor seizure control. Contrast-enhancing gliomas were associated with shorter overall survival times. These data provide additional new information on progression and prognosis based on MRI features in dogs with suspected brain gliomas. This may assist clinicians in establishing a more individualised anti-epileptic treatment, and help owners make the most optimal decisions.

## Figures and Tables

**Table 1:** Classification of neurological findings in 60 dogs with presumed brain glioma.

Neurological Findings	Description
Absence	Only seizures.
Mild/Moderate	At least one of the following: mildly altered mentation, head tilt, ambulatory status, any type of ataxia, proprioceptive deficits, and cranial nerve deficits.
Severe	At least one of the following: severe obtundation, stupor or coma, or non-ambulatory tetraparesis.
Post-ictal	At least one of the following: altered mental status, compulsive behaviour, tetraparesis or paraparesis, bilateral symmetrical proprioceptive deficits, or bilateral menace response deficits.

**Table 2:** Cause of death or euthanasia in 32 dogs with presumed brain glioma located in various rostrotentorial regions of the brain.

Glioma-Related Death of Known Cause	Tumour Location			
	Fronto-Olfactory Cortex (*n* = 14)	Piriform Lobe (*n* = 11)	Temporal Cortex (*n* = 4)	Other Rostrotentorial (*n* = 3)
No improvement—euthanasia within 48 h	2 (14)	0 (0)	1 (25)	0
Progression of neurological signs	9 (64)	1 (9)	2 (50)	1 (33)
Increased intensity of seizures	3 (22)	10 (91)	1 (25)	2 (67)

## Data Availability

The data presented in this study are available on reasonable request from the corresponding author.

## Supplementary Materials

The following supporting information can be downloaded at: https://www.mdpi.com/article/10.3390/vetsci9060257/s1, Table S1: Magnetic resonance imaging (MRI) sequence variable ranges for the transverse T2w, T1w and FLAIR sequences included in this study; Table S2: Demographic and clinical characteristics of the study population; Table S3: Magnetic resonance imaging features of 60 suspected gliomas; Table S4: Univariable analysis of the relationship between demographic & clinical characteristics and MRI location. Table S5: Univariable analysis of the relationship between demographic & clinical characteristics and MRI-based high grade (all 60 dogs included); Table S6: Univariable survival analysis including 48 dogs with rostrotentorial gliomas; Table S7: Multivariable survival analysis including 48 dogs with rostrotentorial gliomas.

## Abbreviations

SE	*Status epilepticus*
CE	Contrast enhancement
CI	Confidence interval
CS	Cluster seizures
CSF	Cerebrospinal fluid
CT	Computed tomography
ES	Epileptic seizures
FLAIR	Fluid-attenuated inversion recovery
HR	Hazard ratio
IDH	Isocitrate dehydrogenase
IQR	Interquartile range
MST	Median overall survival time
MRI	Magnetic Resonance Imaging
OR	Odds ratio
OS	Overall survival time
T1W	T1-weighted
T2W	T2-weighted
T2*	T2-weighted gradient echo
