# Supplementary material for: Clinical and Magnetic Resonance Imaging (MRI) Features, Tumour Localisation, and Survival of Dogs with Presumptive Brain Gliomas
Source: Vet Sci. 2022 May 27;9(6):257. doi: 10.3390/vetsci9060257 (PMC9230849; doi:10.3390/vetsci9060257)
Supplement: Supplementary file 1 [file vetsci-09-00257-s001.zip › vetsci-1655024-supplementary.pdf]

Tables & supplementary files & figures

Supplementary: Table S1. Magnetic resonance imaging (MRI) sequence variable ranges for the transverse T2w, T1w and FLAIR sequences included in this study.

MRI sequence	Repetition time	Echo time	Flip angle	Slice thikeness	Matrix size	Echo train
Transverse T1	399-655	7,5-11	90	3-3,5	224/192-304/256	2
Transverse T2	4000-7500	105-120	90	3-3,5	256/256-320/320	15-23
Transverse FLAIR	7000-8000	120	90	3-3,5	192/160-288/256	19

Supplementary: Table S2. Demographic and clinical characteristics of the study population

**Demographic characteristics**	Number (%) of dogs unless otherwise stated
Sex	
Male neutered	19 (32)
Male entire	13 (22)
Female spayed	23 (38)
Female entire	5 (8)
Male	32 (53)
Female	28 (47)
Male-to-female ratio (CI 95%)	1.14 (0.69 – 1.89)
Entire	18 (30)
Neutered	42 (70)
Entire male-to-female ratio (CI 95%)	2.60 (0.97 – 7.00)
Neutered male-to-female ratio (CI 95%)	0.83 (0.45 – 1.50)
Age [years]	9, 7 – 10 (4 – 15) [a]
Crossbreed	5 (8)
Pedigree	55 (92)
Breeds (of 55 pedigree dogs)	
French Bulldog	40 (73)
Boxer	6 (11)
Yorkshire Terrier	2 (4)
English Bulldog	1 (2)
Dogue de Bourdeaux	1 (2)
Boston Terrier	1 (2)
Cavalier King Charles Spaniel	1 (2)
West Highland White Terrier	1 (2)
Staffordshire Bull Terrier	1 (2)
Cane Corso	1 (2)
Body weight [kg]	14, 12 – 18 (4 – 55) [a]
Body size	
Miniature (< 5 kg)	2 (3)
Small (5 – 15 kg)	30 (50)
Medium (15 – 25 kg)	16 (27)
Large (25 – 40 kg)	8 (13)
Giant (> 40 kg)	4 (7)
Skull shape (of 55 pedigree dogs)	
Brachycephalic	52 (95)
Mesocephalic	3 (6) [2 Yorkshire terriers and 1 WHWT]
Dolichocephalic	0

**Clinical characteristics**	Number (%) of dogs unless otherwise stated
Time to diagnosis [days]	13, 3 – 35 (1 – 730) [a]
Treatment	
Palliative	56 (93)
Definitive	4 (7)
Reason for presentation	
Epileptic seizures (ES)	38 (63)
Epileptic seizures (ES) & neurological signs	8 (13)
Sole neurological signs	13 (22)
Incidental	1 (2)
Epileptic seizures (ES) as clinical manifestation	46 (77)
Cluster seizures/status epilepticus (CS/SE) as the first manifestation of disease	25 (42)
Neurological deficits at examination	31 (52)

[a] median, IQR, and min – max range

Supplementary: Table S3. Magnetic resonance imaging features of 60 suspected gliomas.

**MRI Criteria**	**Description used in the study (number of dogs)**
**Origin**	
Hemispheric (n=53)	Fronto-olfactory (n=20); Piriform (n=18); Temporal (n=10); Parietal (n=3); Occipital (n=1); Ventricle (n=1)
Diencephalon (n=4)	Thalamus (n=4)
Infratentorial (n=3)	Cerebellum (n=1); Brainstem (n=2)
**Margins**	Ill-defined (n=5); Poorly-defined (n=39); Well-defined (n=16)
**Suspected peritumoral edema**	Peritumoral (n=34); Vasogenic (n=4); Vasogenic&peritumoral (n=14); Not assessable (n=3); None (n=5)
**Mass effect**	None (n=22)
Brain herniations	None (n=42); Foramen magnum (n=1); Caudal transtentorial (n=15); Caudal transtentorial&foramen magnum (n=1); Cranial transtentorial (n=0); Subfalcine (n=1)
Ventricular compression	Yes (n=38); No (n=22)
Midline shift	Yes (n=47); No (n=13)

Syringomyelia	Yes (n=20); No (n=40)
Displacement of internal capsule/corpus callosum/hippocampus	Yes (n=46); No (n=14)
**Spread**	Hippocampus (n=17); Other areas (n=43); No (n=10)
**Predominant signal intensity:**	
T2W	Hyperintense (n=52); Mixed (n=8)
T1W	Hypointense (n=53); Isointense (n=2); Mixed (n=5)
FLAIR	Hyperintense (n=32); Hypointense (n=1); Isointense (n=1); Mixed (n=26)
GRE signal void	Yes (n=21); No (n=39)
Contrast enhancement (CE)	
Grade	Mild (n=17); Moderate (n=23); Severe (n=4); None (n=16)
Pattern	Homogeneous (n=1); Heterogeneous (n=22); Ring enhancement (n=7)
**Bone alterations** (compared to the contralateral bone)	Atrophy (n=16); Expansion&atrophy (n=4); Hyperostosis (n=1); None (n=39)
**Suspected tumor grade**	High (n=31); Low (n=22); Equivocal (n=7)

Supplementary: Table S4. Univariable analysis of the relationship between demographic & clinical characteristics and MRI location. Since there were only 3 dogs with caudotentorial gliomas they were only presented and excluded from statistical analysis:

Variable	MRI location					
	Rostrotentorial (n=57)					Caudotentorial (n=3)
	Fronto-olfactory cortex (n=20)	Piriform lobe (n=18)	Temporal cortex (n=10)	Other rostrotentorial (n=9)	p-value	
Male sex	5 (25)*	10 (56)	9 (90)	6 (67)	0.003	2 (67)
Neutered	16 (80)	14 (78)	4 (40)	5 (56)	0.103	3 (100)
Pedigree	19 (95)	17 (94)	10 (100)	7 (78)	0.288	2 (67)
Bulldogs (French and English)	15 (79)	12 (71)	7 (70)	6 (86)	-	1 (50)
Boxer	1 (5)	3 (18)	1 (10)	0	-	1 (50)
Other breeds	3 (16)	2 (12)	2 (20)	1 (14)	-	0
Age [years]	8, 7 – 11 (4 – 15)	9, 7 – 11 (7 – 13)	9 (6 – 11)	8, 7 – 10 (5 – 12)	0.770	5 – 8
Body weight [kg]	12, 10 – 15 (4 – 32)*	16, 14 – 27 (13 – 50)	15, 14 – 20 (4 – 49)	15, 10 – 18 (5 – 42)	0.029	13 – 55

Body size					0.353	
Small & miniature (<15 kg)	15 (75)	8 (44)	4 (40)	4 (44)	-	1 (33)
Medium (15-25 kg) (n=16)	4 (20)	6 (33)	3 (30)	3 (33)	-	0
Large & giant (≥25 kg) (n=12)	1 (5)	4 (22)	3 (30)	2 (22)	-	2 (67)
Time to diagnosis [days]	14, 5 – 41 (2 – 730)	3, 3 – 25 (2 – 212)	15, 3 – 61 (1 – 670)	15, 10 – 31 (1 – 184)	0.549	31 – 57
Seizures	16 (80)	18 (100)*	7 (70.0)	5 (55.6)	0.010	0
Cluster seizures/epileptic status as first manifestation of disease	7 (35)	12 (66.7)	3 (30.0)	3 (33.3)	0.129	0
Neurological deficits at examination	11 (55)	4 (22)*	6 (60)	7 (78)	0.024	3 (100)
Hippocampus affected	0	15 (83)*	1 (10)	1 (11) [ventricular]	<0.001	0

* significant at α=0.1 in pairwise comparisons and included in the multivariable analysis

Supplementary: Table S5. Univariable analysis of the relationship between demographic & clinical characteristics and MRI-based high grade (all 60 dogs included):

Variable	Category	No. (%) of dogs with high grade in the category unless otherwise stated	OR (CI 95%)	p-value
Age [years]	High-grade (n=31)<br>Other (n=29)	8, 7 – 10 (4 – 15) [b]<br>9, 8 – 11 (5 – 12) [b]	-	0.166
Male sex	Yes (n=32)<br>No (n=28)	14 (44)<br>17 (61)	0.50 (0.18 –1.41)	0.188
Neutered	Yes (n=42)<br>No (n=10)	21 (50)<br>10 (56)	0.80 (0.26 – 2.43)	0.693
Pedigree	Yes (n=55)<br>No (n=5)	29 (53)<br>2 (40)	1.67 (0.26 – 10.8)	0.666F
Breeds	Bulldogs (n=41)<br>Boxer (n=6)<br>Others & crossbreeds (n=13) [a]	23 (56)<br>1 (17)<br>7 (54)	-	0.171
Boxer	Yes (n=6)<br>No (n=54)	1 (17)<br>30 (56)	0.16 (0.02 – 1.46)	0.098F*
Body weight [kg]	High-grade (n=31)<br>Other (n=29)	14, 10 – 16 (4 – 42) [b]<br>15, 14 – 27 (5 – 55) [b]	-	0.016*
Size	Small & miniature (< 15 kg) (n=32)<br>Medium (15-25 kg) (n=16)	19 (59)<br><br>9 (56)	-	0.108

	Large & giant (> 25 kg) (n=12)	3 (25)		
Large size (≥25 kg)	Yes (n=12)<br>No (n=48)	3 (25)<br>28 (58)	0.24<br>(0.06 – 0.99)	0.036*
Time to diagnosis [days]	High-grade (n=31)<br>Other (n=29)	20, 6 – 41 (1 – 730) [b]<br>5, 3 – 31 (1 – 670) [b]	-	0.106
Seizures	Yes (n=46)<br>No (n=14)	23 (50)<br>8 (57)	0.75<br>(0.22 – 2.51)	0.639
Cluster seizures/epileptic status as first manifestation of disease	Yes (n=25)<br>No (n=35)	10 (40)<br>21 (60)	0.44<br>(0.16 – 1.27)	0.125
Neurological deficits at examination	Yes (n=31)<br>No (n=29)	20 (65)<br>11 (38)	2.98<br>(1.04 – 8.51)	0.038*
Location	Fronto-olfactory cortex (n=20)<br>Piriform lobe (n=18)<br>Temporal cortex (n=10)<br>Other rostrotentorial regions (n=9)[a]<br>Caudotentorial regions (n=3)	18 (90)<br>2 (11)<br>4 (40)<br>7 (78)<br>0	-	<0.001*
Fronto-olfactory cortex	Yes (n=20)<br>No (n=40)	18 (90)<br>13 (33)	18.7<br>(3.76 – 92.9)	<0.001*
Piriform lobe	Yes (n=18)<br>No (n=42)	2 (11)<br>29 (69)	0.06<br>(0.01 – 0.28)	<0.001*
Hippocampus affected [c]	Yes (n=17)<br>No (n=43)	3 (18)<br>28 (65)	0.12<br>(0.03 – 0.46)	0.001*

[a] reference category; if not stated otherwise, category "No" is always a reference category.
[b] median, IQR, and min – max range
[c] "Hippocampus affected" not included in the multivariable analysis because it seems to limited virtually only to pyriform location
* significant at α=0.1 and included in the multivariable analysis

Supplementary: Table S6. Univariable survival analysis including 48 dogs with rostrotentorial gliomas

Variable	Category	Overall survival time [MST (CI 95%) and IQR [b]]	HR (CI 95%)	p-value
Male sex	Yes (n=25)<br>No (n=23)	-	0.79 (0.41 – 1.51)	0.471
Neutered	Yes (n=33)<br>No (n=15)	-	0.83 (0.41 – 1.70)	0.611
Pedigree	Yes (n=45)<br>No (n=3)	-	1.91 (0.45 – 8.05)	0.378
Breeds	Bulldogs (n=34)<br>Boxer (n=5)<br>Others & crossbreeds (n=9) [a]	-	1.68 (0.67 – 4.19)<br>1.12 (0.31 – 4.02)<br>-	0.269<br>0.857
Age [years]	-	-	1.02 (0.87 – 1.20)	0.807
Body weight [kg]	-	-	0.94 (0.89 – 0.99)	0.018*
Body size	Small & miniature (<15 kg) (n=23) [a]<br>Medium (15-25 kg) (n=15)<br>Large & giant (≥25 kg) (n=10)	-	-<br>0.50 (0.24 – 1.08)<br>0.26 (0.09 – 0.74)	 0.077<br>0.012

Large size (>25 kg)	Yes (n=9)<br><br>No (n=39)	540 (0 – 1260), 14 – UD [c]<br>61 (17 – 104), 31 – 163	0.30 (0.10 – 0.89)	0.031*
Time to diagnosis [days]	-	-	1.01 (0.99 – 1.02)	0.469
≤ 1 week to diagnosis	Yes (n=24)<br>No (n=24)	-	0.62 (0.32 – 1.20)	0.155
Seizures	Yes (n=39)<br>No (n=9)	-	0.73 (0.31 – 1.69)	0.460
Cluster seizures/epileptic status as first manifestation of disease	Yes (n=30)<br>No (n=18)	-	0.63 (0.32 – 1.24)	0.181
Neurological deficits at examination	Yes (n=24)<br>No (n=24)	-	1.81 (0.94 – 3.49)	0.077*
Location	Fronto-olfactory cortex (n=18)<br>Piriform lobe (n=14)<br>Temporal cortex (n=9)<br>Other rostrotentorial (n=7)[a]	-	3.73 (1.07 – 13.0)<br>1.18 (0.33 – 4.23)<br>1.62 (0.40 – 6.61)<br>-	0.039*<br>0.805<br>0.501
Fronto-olfactory cortex	Yes (n=18)<br>No (n=30)	32 (0 – 69), 2 – 85<br>163 (0 – 335), 43 – 354	3.00 (1.51 – 5.94)	0.002*
Piriform lobe	Yes (n=14)<br>No (n=34)	-	0.52 (0.26 – 1.07)	0.076*
Hippocampus affected	Yes (n=13)<br>No (n=35)	-	0.75 (0.37 – 1.52)	0.419
Contrast enhancement	Yes (n=34)<br><br>No (n=14)	46 (10 – 81), 5 – 104<br>198 (159 – 237), 61 – 540	3.23 (1.46 – 7.20)	0.004*
Herniation	Yes (n=15)<br>No (n=33)	-	1.38 (0.68 – 2.83)	0.372
MRI-based grade	High (n=27)<br>Equivocal (n=3)<br>Low (n=18) [a]	-	2.34 (1.13 – 4.84)<br>1.97 (0.55 – 7.05)<br>-	0.022*<br><br>0.297
MRI-based high grade	Yes (n=27)<br>No (n=21)	43 (9 – 77), 5 – 104<br>180 (25 – 335), 46 – 354	2.11 (1.07 – 4.16)	0.032*

[a] reference category; if not stated otherwise, category "No" is always a reference category.
[b] reported only for categories with significantly different overall survival time
[c] undetermined
* significant at α=0.1 and included in the multivariable analysis

Supplementary: Table S7. Multivariable survival analysis including 48 dogs with rostrotentorial gliomas

Variable	Regression coefficient (SE)	Test statistic	p-value	Hazard ratio (CI 95%)

Contrast enhancement	1.18 (0.41)	8.28	0.004*	3.24 (1.46 – 7.20)
Variables removed from the model:				
Fronto-olfactory cortex	0.70 (0.39)	3.25	0.071	2.01 (0.94 – 4.28)
Neurological deficits at examination	0.54 (0.34)	2.47	0.116	1.72 (0.88 – 3.37)
Large body size (>25 kg)	-0.55 (0.53)	1.08	0.299	0.58 (0.20 – 1.63)
Pyriform lobe	-0.05 (0.49)	0.01	0.920	0.95 (0.36 – 2.49)
MRI-based high grade	-0.05 (0.48)	0.01	0.922	0.95 (0.37 – 2.46)

* significant at $\alpha=0.05$
